# Neuronal Electrophysiological Activities Detection of Defense Behaviors Using an Implantable Microelectrode Array in the Dorsal Periaqueductal Gray

**DOI:** 10.3390/bios12040193

**Published:** 2022-03-25

**Authors:** Botao Lu, Penghui Fan, Yiding Wang, Yuchuan Dai, Jingyu Xie, Gucheng Yang, Fan Mo, Zhaojie Xu, Yilin Song, Juntao Liu, Xinxia Cai

**Affiliations:** 1 State Key Laboratory of Transducer Technology, Aerospace Information Research Institute, Chinese Academy of Sciences, Beijing 100190, China; lslbt96@163.com (B.L.); fan_ph@163.com (P.F.); wangyiding18@mails.ucas.ac.cn (Y.W.); daiyuchuan18@mails.ucas.ac.cn (Y.D.); xiejingyu16@mails.ucas.ac.cn (J.X.); gcyang163@163.com (G.Y.); mofan19@mails.ucas.ac.cn (F.M.); 18668915887@163.com (Z.X.); ylsong@mail.ie.ac.cn (Y.S.); 2 University of Chinese Academy of Sciences, Beijing 100049, China

**Keywords:** fear, implantable MEA, electrophysiology, dorsal periaqueductal gray, 2-MT

## Abstract

Defense is the basic survival mechanism of animals when facing dangers. Previous studies have shown that the midbrain periaqueduct gray (PAG) was essential for the production of defense responses. However, the correlation between the endogenous neuronal activities of the dorsal PAG (dPAG) and different defense behaviors was still unclear. In this article, we designed and manufactured microelectrode arrays (MEAs) whose detection sites were arranged to match the shape and position of dPAG in rats, and modified it with platinum-black nanoparticles to improve the detection performance. Subsequently, we successfully recorded the electrophysiological activities of dPAG neurons via designed MEAs in freely behaving rats before and after exposure to the potent analog of predator odor 2-methyl-2-thiazoline (2-MT). Results demonstrated that 2-MT could cause strong innate fear and a series of defensive behaviors, accompanied by the significantly increased average firing rate and local field potential (LFP) power of neurons in dPAG. We also observed that dPAG participated in different defense behaviors with different degrees of activation, which was significantly stronger in the flight stage. Further analysis showed that the neuronal activities of dPAG neurons were earlier than flight, and the intensity of activation was inversely proportional to the distance from predator odor. Overall, our results indicate that dPAG neuronal activities play a crucial role in controlling different types of predator odor-evoked innate fear/defensive behaviors, and provide some guidance for the prediction of defense behavior.

## 1. Introduction

Fear is a crucial natural defense mechanism of humans and animals for survival and self-protection, which is one of the driving forces of species evolution [1,2]. As a basic emotion, fear can be evoked when the brain perceives the danger to induce animals to produce defense behavior and physiological response, such as escape and freezing, to increase the chance of animal survival. The emotional processing system in the brain controls such fear-related behaviors in animals, and abnormalities of the fear circuitry often result in pathological diseases, such as depression, panic disorder, and post-traumatic stress syndrome [3,4]. Fear can be innate or learned (conditioned) [3,5]. Innate fear, which is in the genes imprinted in the evolution process, essentially stems from the threat of dangerous objects [6]. Organisms often use odor information to mark territory, find food, identify similar species, and avoid natural enemies [6,7]. Therefore, previous studies on the innate fear of rodents mainly used the odors of predator hair, urine, and feces to stimulate rodents to perform innate fear and defensive behaviors [8]. Among them, the most commonly used is 2,3,5-Trimethyl-3-thiazoline (TMT) [9,10] and its derivative 2-methyl-2-thiazoline (2-MT) [11,12] extracted from the excrement of the fox, the natural enemy of rats, which can efficiently induce the instinctive freezing and other defensive behaviors of rats [13].

The fear circuitry components for innate fear include, but are not limited to, the frontal lobe areas, amygdala, hypothalamus, cortex, and continuous strip of midbrain structures [14,15,16,17]. The interaction mechanism between these regions is more complicated. The aqueduct gray matter located in the midbrain plays a role in the fear circuit to express appropriate defensive behaviors for fear and regulate the behavior and movement of animals. It is considered to be the effector of defensive behaviors [18]. Previous studies on the fear circuit of rodents have shown that, in the midbrain periaqueductal gray (PAG), the dorsal periaqueductal gray (dPAG) is mainly in the circuit that regulates innate fear behavior [1,19], and the ventral periaqueductal gray (vPAG) is mainly responsible for coordinating acquired fear behavior [20]. Lesions and stimulation in dPAG affect animals’ defensive response to fear [1]. However, only limited information has been obtained from neuronal recording in the dPAG during defense behaviors evoked by the odor of the predator. At present, studies mainly have focused on dPAG activities in relation to conditions stimulation [21,22,23] or optogenetic activation [24,25]. In addition, the exact role of the dPAG remains to be fully studied in predator odor-induced defense behaviors and the correlation between neuronal electrophysiology and different defense behavior. Studying the relationship between fear behavior and dPAG neural activities has important implications for related fear circuits and the treatment of fear-related diseases [26].

Understanding the neural mechanism under innate fear related behaviors requires monitoring both behavioral pattern and electrophysiological activities. The firing of neurons and the transmission of neural signals between different brain regions are the key features in the process of fear generation. Neuronal recording in awake animals can provide an important insight into the role of the particular brain region of fear circuitry in defense behaviors [27,28]. With the development of micro-electro-mechanical system (MEMS) technology, implantable microelectrode arrays (MEAs) based on the MEMS are widely used in neuroscience research [29,30]. The long-term implanted neural probe is a powerful tool for monitoring the brain activities at the single cell level [31,32].

Compared with traditional microwire electrodes, neuron microelectrode arrays can provide important neural information with high spatial-temporal resolution and high signal-to-noise ratio (SNR) for complex animal behavior in the awake state. [17,33]. Thus, we designed a kind of neuronal microelectrode array based on dPAG shape, which can be implanted for a long time in the dPAG to detect neural electrophysiological signals under the fear of moving rats. The distribution of recording sites covered as many areas of dPAG as possible, which was helpful to detect a wider range of neuronal signals in the target area and avoided severe damage to the brain tissue. We applied the MEA to combined neuronal recordings (field potential and spike signal) in the dPAG before and during the behavioral changes evoked by exposure to 2-MT odor stimuli. Simultaneous monitoring of rat movement also allowed us to relate the timing of changes in motor output associated with defense behaviors to the patterns of neural activity. This allowed us to interrogate the roles of dPAG in relation to motor activity associated with innate fear. We used the designed MEA to simultaneously record the changes of neuronal dynamics in the dPAG and behaviors of rats under the influence of 2-MT (predator odor), and found that dPAG neural activities encoded different aspects of defensive behaviors, including flight and freezing. Among them, the activity of neurons during the Flight was early in action. Moreover, the neurological activity during the Flight period was more active than freezing within this region. The results showed that the MEA with high temporal and spatial resolution can effectively provide an electrophysiological basis for studying predator fear-related circuits and the diagnosis and treatment of fear-related diseases.

## 2. Materials and Methods

### 2.1. Animals

The male Sprague-Dawley rats (250–350 g, 7–12 weeks old) were obtained from Vital River Laboratory Animal Technology Co., Ltd. (Beijing, China). All rats were kept in a 12-h light/dark cycle environment, where food and drinking water were freely available. All animal experiments were conducted with the permission of Beijing Association on Laboratory Animal Care and approved by the Institutional Animal Care and Use Committee at Aerospace Information Research Institute, Chinese Academy of Science (AIRCAS).

### 2.2. Fabrication and Nanomodification of the MEA

**Figure 1:**
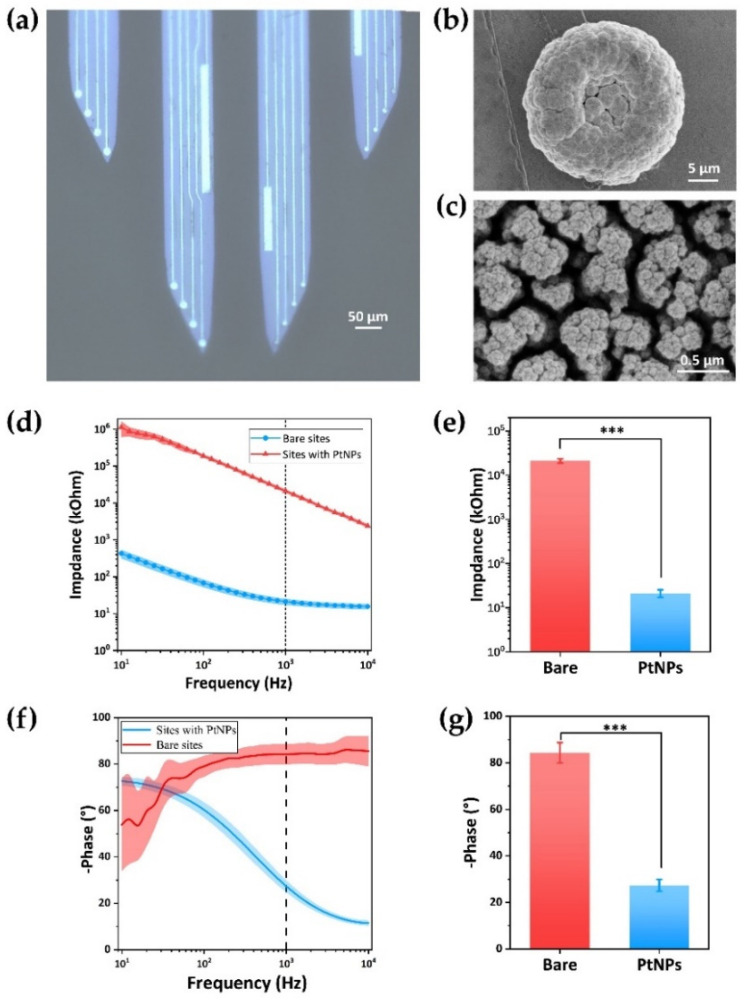
Modification and characterization of MEA. (**a**) Optical image of tip shape of MEA. (**b**,**c**) The morphology of single recording site with black Pt nanomaterials was characterized by a scanning electron microscope (SEM). (**d**) The electrochemical impedance spectroscopy (EIS) and (**f**) phase distribution characterization of recording sites before and after modification of black Pt nanomaterials. (**e**) Average impedance of 16 sites at 1 kHz decreased from 21,147.7 ± 2328.62 kΩ to 21.278 ± 4.080 kΩ (t = 23.97, *** *p* < 0.001). (**g**) The phase changed from −84.29 ± 4.38° to −27.34 ± 2.59° (t = 27.03, *** *p* < 0.001). Results were expressed as mean ± SE. Statistical analysis was performed using Student’s *t*-test.

The microelectrode array was composed of four handles, and each probe has four sites and a counter electrode. We also integrated an array of 16 recording electrodes (diameter = 19 μm) for monitoring the electro-physiological activities of neurons around the probe array. The shape of the MEA was designed in correspondence to the anatomical structure of the dPAG to ensure that all detection sites simultaneously detect neuron signals of dPAG (Figure 1a). Micro-scale silicon has good toughness and biocompatibility and can fully withstand the stress generated by implantation in the brain. Therefore, silicon was selected as the substrate of MEAs [34].The manufacturing process adopts micro-electro-mechanical system (MEMS) technology and silicon-on-insulator (SOI) wafer (25 µm Si, 1 µm SiO_2_, 550 µm Si) was used as the substrate to be processed layer by layer (Figure S1).

First, a 200-nm layer of SiO_2_ was produced for insulating devices from the SOI substrate and AZ 5214-E photoresist was spin-coated to photoetch a conducting layer on the wafer. AZ 5214-E is widely used in the lift-off process because of its high resolution. Then, 250 nm Pt and 30 nm Ti were deposited by sputtering the metal layer. The recording sites, microwires, and banding pads were formed by lift-off. Since the length of the electrode was longer than 1mm, the traditional use of a single material silicon nitride or silicon oxide as the insulating layer would bend due to the internal stress in a single direction. Our insulating layer adopted 500 nm ${\mathrm{Si}}_{3}N_{4}$ and 300 nm ${\mathrm{SiO}}_{2}$, which have opposite stress directions. The process can keep the electrode horizontal without bending due to internal stress. Then, AZ 1500 photoresist was spin-coated on the wafer to selectively etched exposure of recording sites and bonding pads. Finally, the shape of the MEA was formed by deep etching (spin coating AZ 4620 for protection before deep etching). Potassium hydroxide (80 °C, 50%) was used on the back wet etching of the SOI wafer to release MEA [35].

After the electrode was fabricated, we modified the recording site surface of MEAs with platinum nanoparticles (PtNPs) to improve the detection performance of the microelectrode array. The interface modification of the microelectrode array was carried out on an electrochemical workstation (Gamry Reference 600, Gamry Instruments, Warminster, PA, USA). The nanostructure of PtNPs was sequentially formed using the chronoamperometry (CA, −1.1 V, 45 s) in the surface of the recording site, which reduced the variability of sites impedance as much as possible [36]. The PtNPs layer gradually thickens and was easier to peel off with the increase of modification time, resulting in the decline of detection performance in the process of detecting electrophysiological signals. Figure S2 showed that too long electroplating time led to the aggregation of platinum nanoparticles. When the plating time was insufficient, the PtNPs layer would not be dense enough to cover the electrode surface and achieve the ideal electrical performance.

### 2.3. Surgery

Rats were deeply anesthetized by the gas anesthesia machine (RWD520, RWD life science, Shenzhen, China) with 5% isoflurane in oxygen-enriched air, and then fixed into a stereotaxic alignment instruction (51600, Stoelting, IL, USA) with ear rods. During surgery, 1–2.5% isoflurane continued to anesthetize rats. After cutting the scalp and craniotomy, MEAs were implanted into the DPAG using a micro thruster (model 2662, David Kopf Instruments, Tujunga, CA, USA) at a constant implanting speed of 5 μm/s. The location for MEAs implanting was: 6.50 mm posterior to bregma, −0.8 to 0.8 mm lateral to the midline and 5 mm below the cortical surface. To reduce tissue damage and displacement, we used dental cement and 5 stainless steel nails anchored to the skull to fix the detection platform on the head of the rat. In order to ensure a sufficient signal-to-noise ratio (SNR > 3), five stainless steel nails were connected by ground wires and linked to electrodes to lead out of the body. After the operation, the rats were injected with 40,000 units of penicillin to reduce inflammation. Rats were housed for 4–5 days to recover to healthy and active status after surgery.

### 2.4. Behavioral Recording Procedures

**Figure 2:**
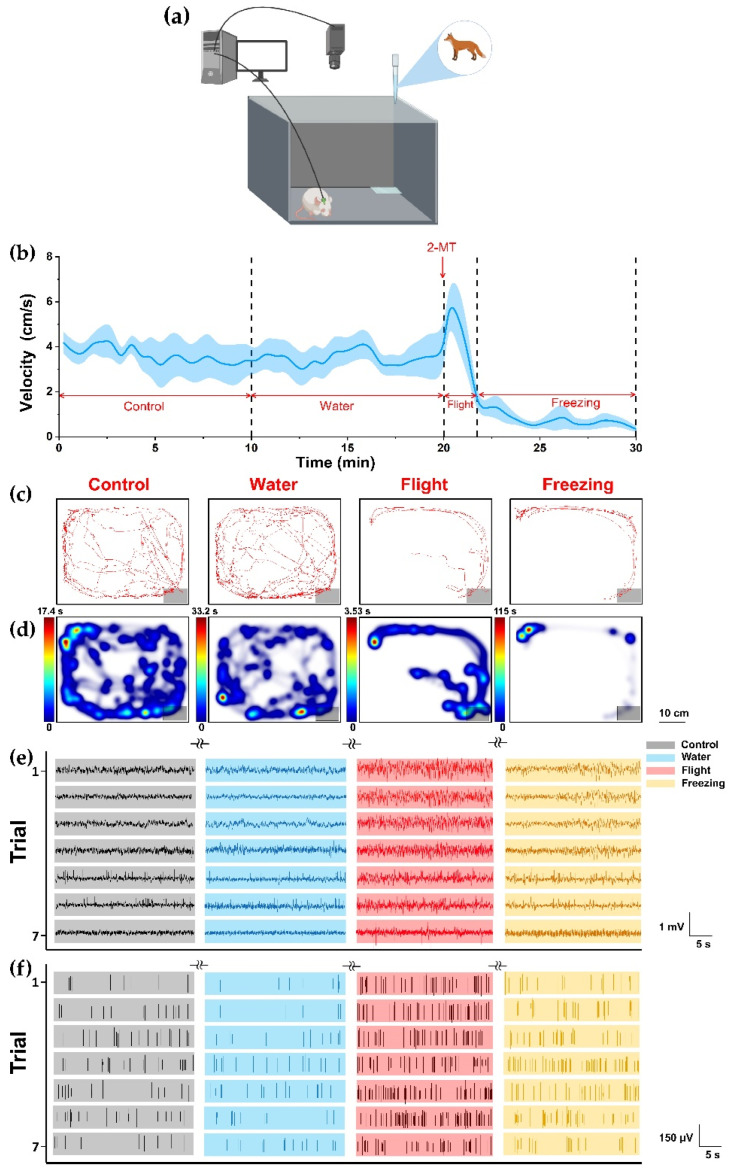
Behavior and electrophysiological activity records of predator odor induced defensive behavior in rats. (**a**) Schematic diagram of innate fear induced by 2-MT in rats; (**b**) average instantaneous velocity of rats in the experiment. The data were the average speed in every 20 s; (**c**) locomotion tracks and (**d**) heatmap of a representative rat in the four states, the gray area indicated the location of the filter paper; (**e**) spike and (**f**) LFP of seven typical channels.

Predator odor potent simulant 2-methyl-2-thiazoline (2MT) was purchased from Shanghai Acmec Biochemical Co., Ltd. (China). In order to analyze the freezing behavior induced by 2-MT, rats were placed in an opening exposure cage (L × W × H = 40 × 50 × 40 cm), and an odorless filter paper was placed in the corner of the field to facilitate the application of each odor (Figure 2a). In our experiment, instead of providing rats with hiding opportunities as in other studies, we used the form of an exposure box without a hiding room, so that rats could not escape from the scope of the experiment and hide, and the most effective defense behaviors were freezing and flight (these two behaviors are also mainly discussed). Therefore, the rat defense stage was mainly composed of escape and freezing in our paradigm. Before the whole experiment, the rats were allowed to freely explore in the cage for 10 min to adapt the experimental environment (once a day, 4–6 days for continuous). Rats were also adapted to 10 min before each test. Each rat was subjected to the test of three phases. In the first phase, as a control group, the rat was allowed to explore freely in the cage for 10 min without external interference. In the second and third phases, 100 μL of deionized water (without 2-MT, water group) and 100 μL of 2-MT were dripped on the filter paper, respectively, and the rat continued to explore freely for 10 min. All tests are collected with a video recording system. Video analysis software Ethovision performed motion analysis of the decoded video to measure speed and spatial coordinates of rats. After each experiment, 75% ethanol was used to completely clean the exposure box, and it is placed in a fume hood for 1 h to prevent odor interference between the experiment. Rats were exposed to each tested odor (water or 2-MT, respectively) only once.

Freezing was a behavior, which was defined as in addition to breathing without other exercise behavior to measure fear. Rats were considered to be in a freezing state if motion was not detected in 2 s (rats in the video in the video, and the previous frame position did not change). The freezing rate is defined as the proportion of the cumulative duration of freezing in the total time.

### 2.5. Histology

After the behavioral tests, rats were deeply anesthetized with 10% chloral hydrate and perfused with 0.9% NaCl and 4% paraformaldehyde in turn. The brain was then removed and placed in 20% sucrose solution at 4 °C to fix for 24 h. After fixation, the brain was cryoprotected in 30% sucrose solution at 4 °C and coronally sectioned at 50 μm. Figure S3 showed the histological validation diagram of the target location. The results showed that MEA of long-term implantation was accurately implanted into the dPAG.

### 2.6. Statistics

All statistics were performed using Origin (OriginLab, Northampton, MA, USA). All data are expressed as mean ± SE unless otherwise specially illustrated. The significance test of different groups were determined using Student’s *t*-test, one-way ANOVA with Tukey’s post hoc tests, or two-way ANOVA with Bonferroni’s post hoc tests.

Neurophysiological signals were captured using a 128-channel neuron data recording system (Blackrock Microsystems, Salt Lake, UT, USA). Neural signals were sampled at 20 kHz. Spikes and the local field potentials (LFPs) signals were extracted by a 200 Hz high-pass and 200 Hz low-pass filter, respectively. Neural Data were analyzed using NeuroExplorer software (Nex Technologies, Colorado Springs, CO, USA). These two kinds of signals carried a lot of information about the activity of neurons in the body. The spike was a high-frequency electrical signal that activates a single neuron. LFP reflected the sum of pulse activities of a large number of neurons, which is closely related to pulse discharge. The local field potential signal is a mesoscopic signal produced by multiple neurons, recording the changes of nerve signal about 200 μm near the microelectrode.

## 3. Results

### 3.1. Characterization of MEAs

Figure 1a displayed the completely clean MEAs consisting of 4 shanks and 16 electrodes distributed on the four tips. The bare electrode was cleared and metallic while it will turn to black after being modified with Pt nanoparticles (PtNPs). Compared with the bare site, the PtNPs detection site was rough, and the particles are densely distributed. The scanning electron microscope (SEM) image (Figure 1b,c) demonstrated uniform dense nanoparticles. The enlarged image after high magnification showed that the PtNP detection site has a complex porous structure, which can increase the contact area and detection sensitivity of the site.

We then evaluated the impedance of the detection sites with the 10–10,000 Hz Electrochemical Impedance Spectroscopy (EIS). The results (Figure 1d,f) showed that the PtNPs modification can effectively reduce the impedance magnitude and improve the phase shift of the detection sites. The average impedance amplitude at 1kHz was reduced from 21,147.7 ± 2328.62 kΩ to 21.278 ± 4.080 kΩ (Figure 1e). At the same time, the phase changed from −84.29 ± 4.38° to −27.34 ± 2.59° (Figure 1g). The impedance curve of the recording sites was smoother, indicating that these sites have better electron transfer ability. The MEAs used in this paper were prepared by the same SOI, and there was little difference in the electrical properties of the recording sites modified with PtNPs in these MEAs (Figure S3). Because the neural signals belong to micro amplitude signals, reducing impedance can improve the SNR of detection sites (Figure S4). The above results showed that the PtNPs-modified microelectrodes have good electrical performance.

### 3.2. Behavioral and Electrophysiological Recording of Pedator Odor Induced Innate Fear

To further understand endogenous neural mechanism of dPAG related to defense behavior, we simultaneously detected electrophysiological signals and behavioral patterns of rats under innate fear (*n* = 10). We found that, after we conducted 2-MT stimulation, the rats suddenly began to move away from the filter paper quickly, and there was an obvious flight behavior. From the point of view of the average instantaneous velocity of the rat during the whole time period, a short peak of movement speed also appeared after exposure to 2-MT (Figure 2a). We considered this stage to be the flight stage. After a short flight period, the movement activity decreases rapidly, and the average movement velocity tended to 0. The rats curled up in the corner and showed strong freezing defense behavior. Flight and avoiding freezing were the typical defensive behaviors of rats with congenital fear. In order to verify the reliability of the results, in addition to the control group, we set up a water group to eliminate the effect of drop behavior on rats in vitro. We counted the locomotion tracks (Figure 2b) and heatmaps (Figure 2c) illustrating the density of the spatial location of representative rats. During control or water, rats actively explored the larger range of space as observed. There was no significant difference in behavior between water group and control group. The results showed that dripping water had no significant effect on the behavior of rats. By contrast, after exposure to 2-MT, rats showed obvious avoidance responses and stayed away from the filter paper with 2-MT, especially freezing. These results confirmed that 2-MT can effectively induce innate fear and defensive behaviors.

We analyzed the neural spikes (Figure 2d) and local field potentials (Figure 2e) of the control, water and 2-MT group (*n* = 5). After exposure to 2-MT (including flight and freezing), LFP waveform of dPAG in rats showed higher frequency and amplitude, and the spike fires were more intensive. On the other hand, the frequency and amplitude of LFP waveform and the release density of spike were almost the same between the control and water group. That is to say, from the electrophysiological signal, the dripping water did not cause dPAG response. These results confirmed that 2-MT could effectively cause innate fear in rats and showed strong defense behaviors (including flight and freezing). At the same time, with the fear state, neurons in the dPAG were significantly activated.

**Figure 3:**
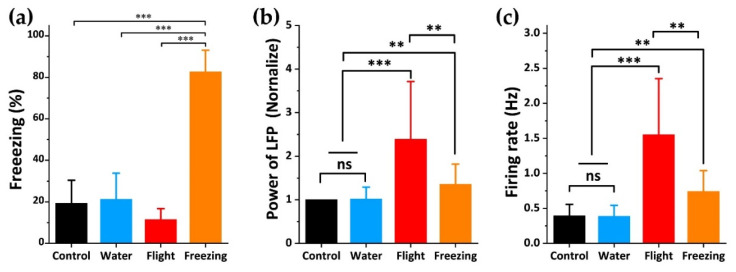
Quantitative analysis of main parameters of behavior and electrophysiology. (**a**) freezing percentage of rats during four different periods; (**b**) normalized power of LFP of rats during four different periods; (**c**) average firing rate of spikes of rats during four different periods. Results were expressed as mean ± SE. Statistical analysis was performed using Student’s *t*-test. ** *p* < 0.01, and *** *p* < 0.001.

**Figure 4:**
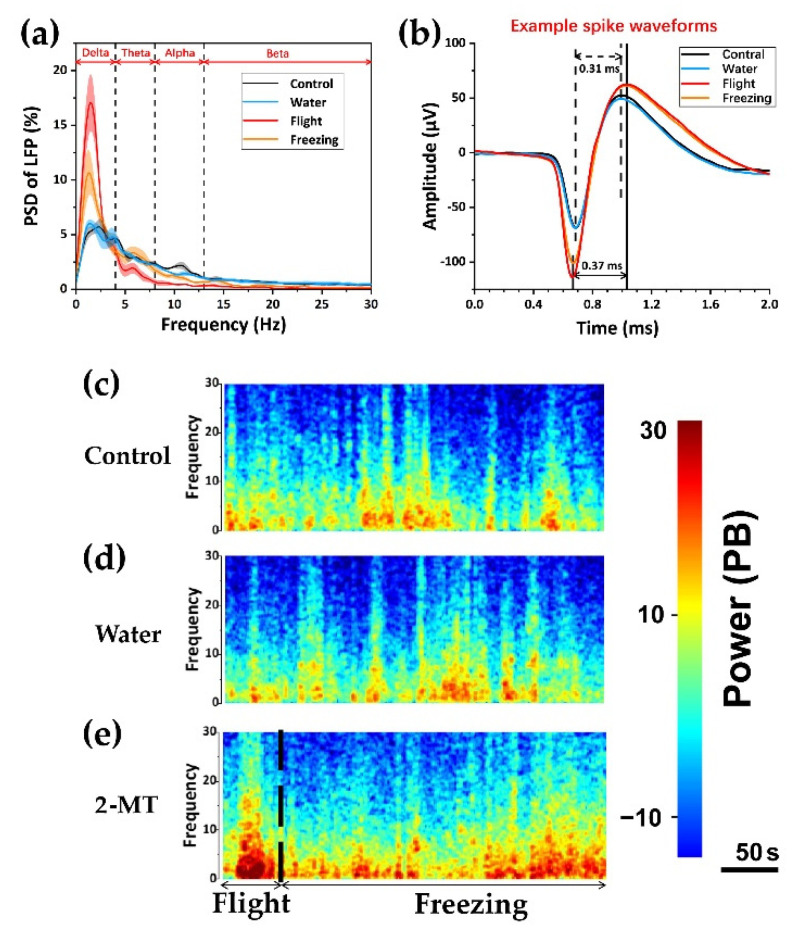
Detailed electrophysiological analysis. (**a**) the power spectral density of LFP in the frequency band from 0 to 30 Hz; (**b**) typical waveforms of spikes during four different periods. Waveform of a single unit clustered from the recording site. (**c**–**e**) representative spectrogram of changes in LFPs during control, water, and 2-MT. After 2-MT dipping, the spectrogram of LFPs increased significantly. The 2-MT state was divided into flight and freezing, and the power spectrum energy was significantly higher during flight. Results were expressed as mean ± SE.

Next, we analyzed the data of rat behavior and electrophysiology. The freezing rate was the main indicator of fear. After the appearance of predator odor, the freezing rate decreased slightly in the flight, and then entered a state of a relatively high freezing rate (Figure 3a). At the mesoscopic level, we normalized the power of LFP to improve the discrimination (Figure 3b). It was found that it was significantly higher during flight than during control and water. In the freezing stage, the power of LFP was lower than during flight, but it was also significantly higher than during control and water. It indicated that, after exposure to predator odor, the neural activity in the dPAG was more intense during flight. This indicates that predator odor activates the dPAG, and outputs of dPAG were strongest during flight. The performance of various parameters during control and water was not different, which further eliminated the possibility of the effect of the liquid itself on rats. At the microscopic level, the firing rate of spike reached the highest level in the flight and decreased in the freezing, but it was still significantly higher than that in the control and water group (Figure 3c). We also counted the average spikes waveform (Figure 4b). After exposure to the odor of the predator, the average amplitude of spike during flight and freezing was higher than that during control and water, and pulse width was slightly increased. Since the power of LFP was mainly concentrated at 0–30 Hz, we divided it into delta frequency band (1–4 Hz), theta frequency band (4–8 Hz), alpha frequency band (8–13 Hz), and beta frequency band (13–30 Hz) (Figure 4a). It can be seen from the figure that, after exposure to predator odor, the proportion of delta frequency band increased significantly, indicating that the activity of dPAG activated field potential caused by predator odor was mainly in the delta frequency band. This can also be reflected in the power spectrum heat map (Figure 4c).

These results confirmed that dPAG was closely involved in regulating odor induced innate fear defense behavior. The neurons in dPAG were significantly activated during exposure to predator odor, and there was no significant change during water and control. In different defensive behaviors, the activation degree was also different, and the activation degree was significantly stronger during flight.

### 3.3. Relationship between dPAG Activity and Flight Behavior during Exposure to Predator Odor

**Figure 5:**
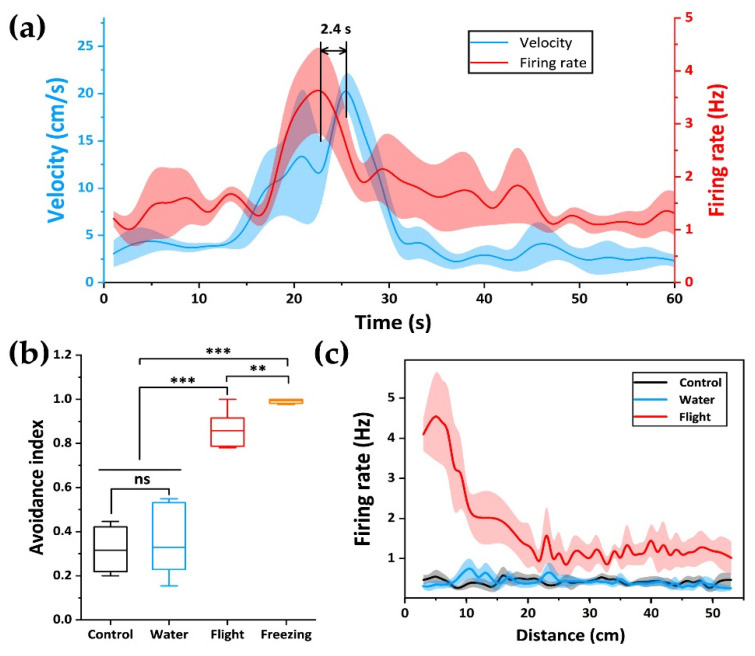
The correlation between neurons and the flight behavior of rats during flight. (**a**) firing activity of neuron and the rats’ instantaneous velocity during flight. The data were the average speed in every 0.5 s; (**b**) avoidance index during four different periods. The middle line of the boxplot was the mean line; (**c**) firing rate plotted as a function of distance to the filter paper during control (black), water (blue) and flight (red). Firing rate was inversely proportional to distance during flight. Results were expressed as mean ± SEM. Statistical analysis was performed using Student’s *t*-test. ** *p* < 0.01, and *** *p* < 0.001.

The use of MEA can monitor the electrophysiological signals of neurons in real time and display the changes of dynamic neural signals. To further analyze the correlation between dPAG neurons and flight behavior, we used MEA and a video recording system to map the real-time changes of instantaneous velocity and spike fire during flight (Figure 5a). We found that the peak of spike fire appeared before the peak of instantaneous velocity, with an advance time of about 2.4 s. This revealed the regulatory effect of dPAG on flight behavior in the fear circuitry, and dPAG had neurons related to flight.

After exposure to predator odor, we found that rats tended to stay away from odor sources (filter paper). In order to explore the occurrence of this behavior, we defined an avoidance index (Figure 5b and Figure S5) to divide the whole space into four quadrants. The time spent in the three quadrants other than the filter paper minus the percentage spent in the quadrant where the filter paper was located, the closer the value was to 1. It meant that the rats would avoid approaching the odor emitting area as much as possible. It can be seen that the escape coefficient of rats during freezing is close to 1, indicating that rats were eager to stay away from odor sources. At the same time, we also plotted the relationship between the distance from the center of the filter paper and the release rate of neuronal action potential (Figure 5c). dPAG cells close to the odor source showed obvious emission. As the rats fled farther, the firing rate decreased gradually. It was basically inversely proportional to the distance from the odor source. In order to determine whether this phenomenon was caused by interference related to defense behavior itself or something unrelated (such as nonspecific motor activity of rats), we also mapped the relationship between distance and action potential release rate in control group and water group. We did not find a discharge pattern similar to flight.

## 4. Discussion

Innate fear is induced by conserved dangers among species and is acquired over the course of evolution. In this article, based on the shape of dPAG, we designed a neural microelectrode array that can be used to synchronously detect the electrophysiological signals of neurons in the fear state of freely moving rats, and successfully detected the fear nerves induced by 2-MT in rats. As a potent analog of predator odor, 2-MT can cause a higher freezing rate than the common predator odor TMT, and the fear effect was more significant. Activities of dPAG neurons were cross-correlated with the behavioral response of rats. By electrophysiological and video recording, we showed that 2-MT activates dPAG cells to induce defense responses (including flight and freezing). The PSD of LFP contains a lot of information about nucleus activities. The proportion of delta waves in the power of LFP was significantly higher during flight and freezing, and reached the highest during flight. Brain regions that are “used” more show higher slow wave activities than those that are less engaged areas [37]. The augmentation of delta wave after rats were exposed to predator odor suggested that dPAG was highly active in the process of defense behavior in rats. The highest proportion during flight indicates that dPAG was used more in this period. Spike signal directly reflected the activities state of a single neuron. dPAG neurons not only increased spike firing rate, but also changed spike waveform in the defense state of rats. The reduction of spike duration and increase of amplitude indicated that the interaction between neurons was swifter, which may be correlated with the high-firing pattern of neurons during defense behavior. Our results suggested that dPAG neurons were closely involved in predator odor induced flight behavior. These neurons were strongly activated after the brain sensed the smell of predators, resulting in flight behavior in rats. The degree of activation was related to the distance from the predator during flight. In addition, its neuronal activity was several seconds earlier than the flight behavior, indicating that the activity of dPAG can also reflect whether there was a potential defense state in rats, which can provide theoretical guidance for the prediction of defense behavior in advance.

Studies of neural activity in central circuits associated with fear have focused on the amygdala. It was generally considered that the direct amygdala–PAG pathway is inhibitive, and it has been predicted that target neurons in PAG will reduce their activity during fear behavior [28]. Our results indicate that the dPAG cells increase their activity during fear, which means that the main impact of almond nucleus on PAG is the effect of inhibiting inter neurons, leading presumably to disinhibition of other PAG cells [22,38]. After exposure to the predator odor, there was widespread activation of PAG cells, as measured by c-fos [39]. The general understanding of the role of PAG in defense behaviors was that the dorsal PAG activity is mainly associated with the escape and flight, while the ventral PAG activity was responsible for freezing [22,38,40]. This is also consistent with our results. We also found that these flight related neurons were also continuously activated during freezing, but the degree of activation was lower than during flight. Flight related neurons in dPAG may participate in freezing and flight behavior at the same time, but they produced different behavior due to different activation degree. The changes of electrophysiological information, combined with behavioral analysis of dPAG neuro-electrophysiological signals in different defense behavior states, provide an electrophysiological basis for studying predator fear-related circuits. dPAG neurons in the flight phase increased their firing rate more than in the freezing phase in response to exposure to the predator odor. Our research also revealed some undescribed features of dPAG and detected the advancement of dPAG brain regions that control flight behavior at the level of neural action potential firing, and their peak activity is also related to the distance from the odor source. In the following research, to better understand the processing process of predator-fear at the circuit level, we will study the situation in which other areas within the PAG participate in predator fear, as well as their internal information transmission direction, and explore the input and output of different areas of the PAG.

## 5. Conclusions

In the present study, a neural microelectrode array was designed and fabricated to detect the dynamic neurophysiological activities of dPAG region in freely moving rats under different defense states. The results showed that dPAG participated in different defense behaviors with different degrees of activation, which was significantly stronger in the flight stage. Further analysis showed that the neuronal activities of dPAG neurons were earlier than flight. Our study revealed the cellular mechanism of dPAG in different defense States, which laid a foundation for the prediction of defense behavior and the treatment of related diseases in the future.

## Data Availability

Data are contained within the article.

## Supplementary Materials

The following supporting information can be downloaded at: https://www.mdpi.com/article/10.3390/bios12040193/s1, Figure S1: Fabrication of the MEA, Figure S2: Scanning electron microscope (ESM) characterization of platinum nanomaterials (PtNPs), Figure S3: Location of microdamage caused by long-term implantation of electrodes in the brain, Figure S4: Electrical performance characterization of different MEAs in the same batch, Figure S5: Innate fear induced by 2-MT in rats.
