# Supplementary material for: Neuronal Electrophysiological Activities Detection of Defense Behaviors Using an Implantable Microelectrode Array in the Dorsal Periaqueductal Gray
Source: Biosensors (Basel). 2022 Mar 25;12(4):193. doi: 10.3390/bios12040193 (PMC9032743; doi:10.3390/bios12040193)
Supplement: Supplementary file 1 [file biosensors-12-00193-s001.zip › biosensors-1615507-supplementary.pdf]

biosensors

MDPI

Article

Supplementary information: Neuronal Electrophysiological Activities Detection of Defense Behaviors Using an Implantable Microelectrode Array in the Dorsal Periaqueductal Gray

Botao Lu [1,2], Penghui Fan [1,2], Yiding Wang [1,2], Yuchuan Dai [1,2], Jingyu Xie [1,2], Gucheng Yang [1,2], Fan Mo [1,2], Zhaojie Xu [1,2], Yilin Song [1,2], Juntao Liu [1,2,*] and Xinxia Cai [1,2,*]s follows:

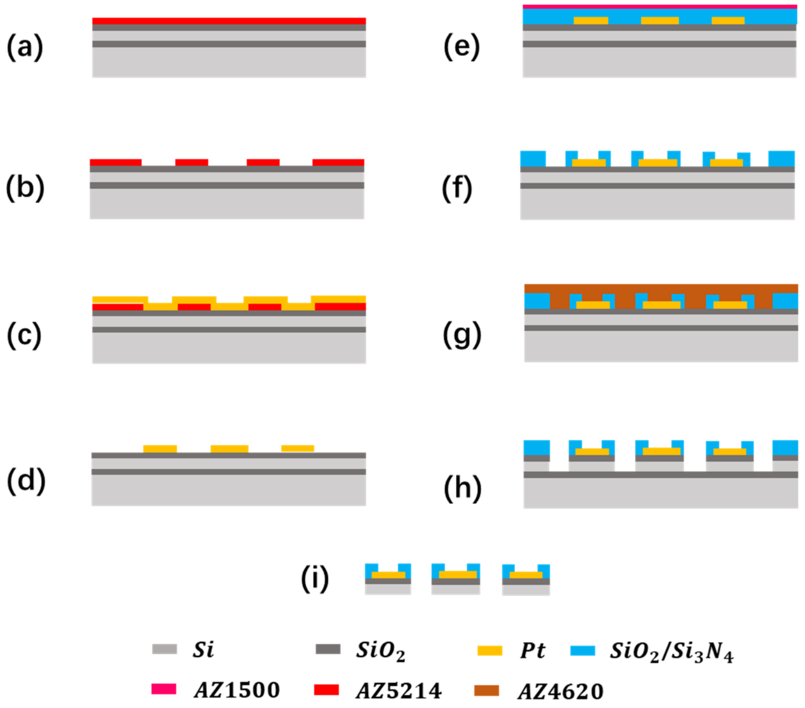

Figure S1 Fabrication of the MEA. (**a**) A 200-nm layer of $SiO_2$was produced for insulating devices from the SOI substrate and AZ 5214E photoresist was spin coated on wafer. (**b**) AZ 5214 photoresist patterned after exposure and development. (**c**) 250 nm Pt and 30 nm Ti were deposited by sputtering as metal layer. (**d**) Sputtering and lift-off to form the metal Pt/Ti layer. (**e**) 300 nm SiO_2 and 500 nm Si_3N_4 was deposited as insulating layer and AZ 1500 photoresist was spin coated on wafer. (**f**) Exposure of recording sites and bonding pads. (**g**) AZ 4620 photoresist was spin coated on wafer and patterned after exposure and development. (h)The MEA probe shape was format. (**i**) Release of the MEA from the substrate.

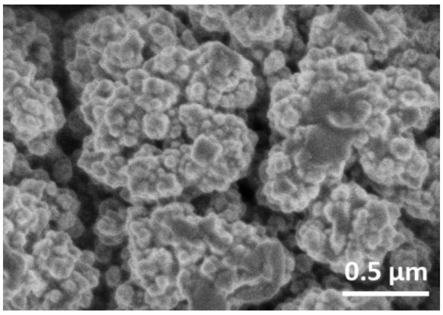

Figure S2 Scanning electron microscope (ESM) characterization of platinum nanomaterials (PtNPs). The morphology of single recording site with PtNPs was characterized using chronoamperometry (CA, -1.1V, 70s). Obvious aggregation of PtNPs was seen.

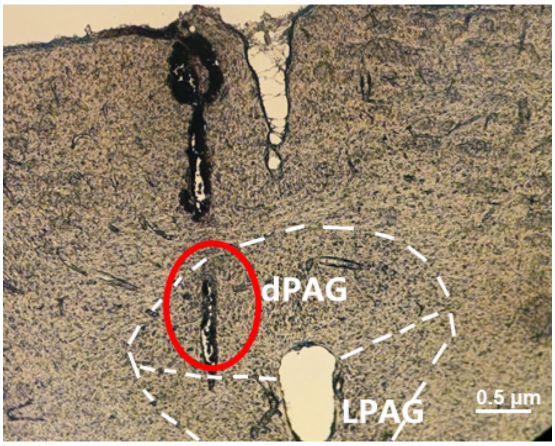

Figure S3. Location of microdamage caused by long-term implantation of electrodes in the brain.

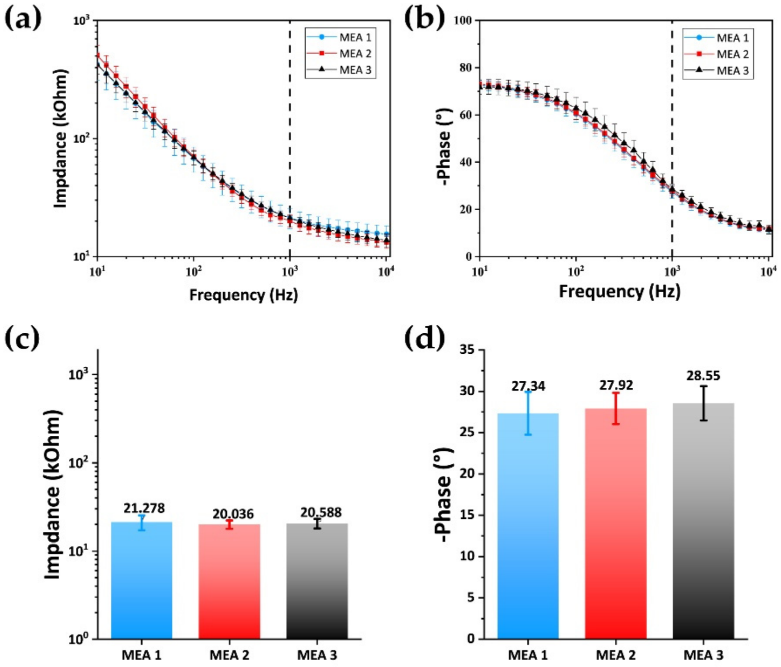

Figure S4 Electrical performance characterization of different MEAs in the same batch. (**a**)Impedance characteristics and (**b**) phase distribution characterization of different MEAs modified with PtNPs. (**c**) Impedance of different MEAs at 1 kHz. MEA 1: 21.278 ± 4.080 kΩ, MEA 2: 20.036 ± 2.150 kΩ and MEA 3: 20.588 ± 2.583 kΩ. (**d**) Average phase of different MEAs at 1 kHz. MEA 1: 27.34 ± 2.59 °, MEA 2: 27.92 ± 1.89 ° and MEA 3: 28.55 ± 2.08 °. 16, 14, and 16 recording sites of MEA 1-3 were successfully modified.

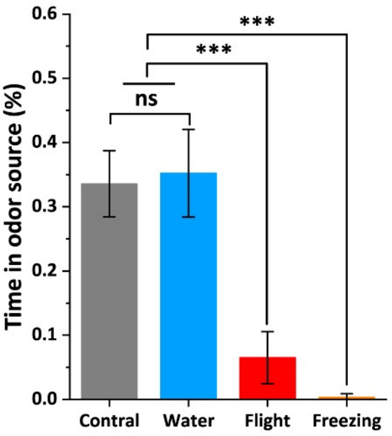

Figure S5. Innate fear induced by 2-MT in rats. The exposure box space was divided into four quadrants, and the rats spent time in the area of predator odor in four stages. The rats in flight and freezing stage were more inclined to the quadrant of the predator odor source. Statistical analysis was performed using Student's t-test. ***$p < 0.001$.
